# Forced migration and human trafficking: survivors’ narratives of strength and survival upon first arrival in Germany

**DOI:** 10.1186/s12939-026-02875-2

**Published:** 2026-05-08

**Authors:** Estella Alejandra Tambini Stollwerck, Irja Rzepka-Marot, Hans-Christoph Friederich, Christoph Nikendei

**Affiliations:** Department for General Internal Medicine and Psychosomatics, Heidelberg University Hospital, Im Neuenheimer Feld 410, 69120 Heidelberg, Germany

**Keywords:** Human trafficking, Trafficking in human beings, Exploitation, Refugees, Qualitative research, Resilience

## Abstract

**Background:**

Human trafficking poses a major public health challenge to the international community, with significant health and social consequences for those affected. Forced migrants are particularly vulnerable to becoming victims of human trafficking due to language barriers and migration-related hardships. These include social and economic deprivation. To better understand the lived experiences within this already vulnerable group, it is essential to examine individual cases in relation to risk factors, experiences of exploitation and exit, and resilience.

**Methods:**

Using a qualitative approach, this study examines social determinants and risk factors of forced migrants who survived human trafficking. It explores their experiences, eventual escape, and sheds light on their resilience. For this purpose, semi-structured interviews regarding the trafficking experiences were conducted with newly arrived forced migrants at a reception and registration centre in Germany. The presence of human trafficking was determined through an initial screening procedure and then confirmed in a personal interview. Additionally, we assessed the refugees’ mental health burden with brief screening questionnaires for post-traumatic stress disorder, depression, anxiety, and the overall stress level (PC-PTSD-5, GAD-2, PHQ-2, RHS-15 distress thermometer).

**Findings:**

A total of 20 interviews were conducted with 14 female and 6 male participants. The participants came from 9 different countries. Most of them experienced sexual exploitation (*N* = 11), labour exploitation (*N* = 7). Few were trafficked but not exploited (*N* = 3). Participants reported that financial hardship was the main benefitting exploitation risk, and in many cases, they had been recruited by individuals they already knew. Spiritual rituals were sometimes used to increase pressure and control. Various forms of violence were inherent to the trafficking situations. In most cases, those affected managed to free themselves. Interpersonal connections and religious beliefs played a crucial role in coping with these experiences, however the screening for common mental disorders among refugees still indicated high levels of psychological distress. The results are discussed in relation to existing literature and implications for support and intervention are presented.

## Introduction

Human trafficking is a profound human rights issue. According to the Palermo Protocol from 2000 “trafficking in persons” is defined as the recruitment, movement or harbouring of persons by deception, the use of threats, coercion or the abuse of a position of vulnerability for the purpose of exploitation. This definition also explicitly states that the consent of the victim does not count as voluntary if violence, coercion, deception, or similar means were used [1]. Human trafficking includes acts of forced labour, sexual exploitation, slavery or servitude, forced criminal activity as well as the removal of organs and forced marriage [1, 2]. Individuals can be recruited by agencies of any kind, whether they operate legally or illegally, or are part of organized criminal networks [3]. The decisive motive is the greatest possible profit, meaning that an individual’s emergency situation, be it economic in nature, political instability in the country, or marginalization, is exploited [4]. It is an almost invisible crime, especially with technological changes that facilitate anonymous contact with potential victims [5]. Any international crisis or local conflict is likely to increase the amount of trafficked persons and climate change is multiplies trafficking risks due to crop loss or weather-induced disasters [5].

Experiences of human trafficking are frequently associated with significant health consequences for the affected individuals. The impacts on both mental and physical health interact with and are shaped by the conditions inherent to trafficking situations [6]. Due to the complexity of the health consequences, including the risk of chronicity human trafficking constitutes a significant global health concern [7]. According to estimates, around 50 million people worldwide are affected [8].

Forced migrants are particularly vulnerable to becoming victims of human trafficking as acute crises, war, and national disasters, often require people to flee quickly with few resources. Moreover, forced migrants often have limited knowledge on local customs, labour market regulations, or workers’ rights. They also often lack legal status or work eligibility, making them more vulnerable to false promises about jobs and quick opportunities more easily to generate money [8–10]. Traffickers take advantage of migrants’ distance to their social network, language barriers and socioeconomic hardship [11]. This frequently occurs along the major migration routes, such as North Africa prior to crossing the Mediterranean, or from East Africa toward the Arabian Peninsula [12].

Risk factors to become a victim of human trafficking for forced migrants are multisystemic. Structural vulnerabilities include lack of legal documentation, prolonged or secondary displacement, and restrictive migration policies, which can constrain access to protection and resources [9]. Individual-level factors, such as prior experiences of exploitation, minority status, or substance dependence, further increase vulnerability [9]. A study of Ethiopian returnees showed that also strong hopes for better living conditions and glamorous descriptions of life abroad, as well as hopelessness about success in their own country, also contributed to the risk of becoming victims of human trafficking [13]. Human trafficking can occur in different stages of flight: trafficking can precede displacement, when individuals initially find themselves in a trafficking situation, from which they subsequently attempt to escape. During their escape, they may become victims of human trafficking, for example due to financial hardship or limited opportunities to protect themselves. Aswell in settings such as large asylum centres or refugee camps, where many people are forced to live together for long periods of time, people can become victims of human trafficking [14]. In general, individual vulnerability is substantially amplified through the intersection of multiple risk factors and unfavourable social determinants, highlighting the structural dimensions of trafficking vulnerability [9]. Above all, the fact that forced migrants often have to remain in circumstances of legal, financial and social uncertainty for long periods of time increases the risk of revictimization [15].

Yet, vulnerability does not preclude survivors’ resilience. Resilience can generally be described as the ability to overcome adversity [16]. Windle, [17] defines resilience as ‘the process of effectively negotiating, adapting to, or managing significant sources of stress or trauma. Assets and resources within the individual, their life and environment facilitate this capacity for adaptation and ‘bouncing back’ in the face of adversity’ ([17]; p. 12). It is shaped by a range of factors, encompassing educational and occupational status, interpersonal dimensions such as marital status, social networks, and social inclusion [18], as well as intrapsychic resources including sense of coherence [19]. In a review on resilience among trafficking survivors, Knight et al. [20] identified that resilience in survivors of human trafficking is comparable to other populations who have experienced victimization. Two recent publications asked survivors of sex trafficking about their concept of resilience and their knowledge on promoting resilience [21, 22]. Participants were asked what the term meant to them, whether they could provide personal examples of how they manage challenges, and what advice they would offer other survivors to foster resilience. On the one hand, they describe a connection to themselves and to others, which they were able to experience particularly through acts of kindness or by supporting other affected individuals. Through this, they were able to have experiences beyond those related to trafficking and to feel positive emotions as well as a sense of meaning [21]. Furthermore, they described an inner sense of resistance—a deliberate decision not to internalize the horrific experiences or allow themselves to be broken or defined by them. Resilience was further conceptualized not as a static state, but as an ongoing process or journey, involving the development from adverse circumstances toward a more positive future, while continually facing challenges and advocating for oneself along the way [22]. Yet, there has been little research into resilience among forced migrants who have been victims of human trafficking.

In order to better understand and analyse the phenomenon of human trafficking among forced migrants and further to provide survivors with adequate support, it is particularly important to listen to the individual stories. Drawing on the definition of resilience described by Windle [17], this study seeks to:

1. Identify common social determinants and risk factors for human trafficking within a sample of forced migrants in an initial reception center in Germany.
2. Explore individual experiences of recruitment, trafficking, and the circumstances surrounding escape, drawing on qualitative insights.
3. Examine mechanisms of resilience among survivors.

## Methods

### Study design and ethical considerations

This study employs a qualitative research design utilizing semi-structured interviews. All participants were identified as survivors of human trafficking in a German reception and registration centre. The study was approved by the local ethics committee (Ethics Committee, Medical Faculty of Heidelberg University, S249/2021; 05.05.2021) and was in line with the Declaration of Helsinki.

### Setting

The Patrick Henry Village Heidelberg (PHV) is a reception and registration centre where asylum-seekers are registered in the state of Baden-Württemberg, Germany. It accommodates around 1000 to 3000 newly arrived asylum-seekers. During their stay at the PHV, state employees verify their identity and register their personal data as a part of the asylum procedure. A psychosocial walk-in clinic, medical support services, and local counselling services are available to all asylum-seekers [23–25].

### Participants

The participants were selected via two different ways: The first (Group A) involved a pre-selection by psychologists, midwives, and psychiatric doctors working in the medical walk-in clinic of the reception and registration centre [24] that had been informed about human trafficking and the performed study. The medical staff informed the researchers about refugees who potentially met criteria of human trafficking, as they answered one of the following questions with ‘yes’ (N = 10) [26]:

- Have you ever been in a situation where you (or anyone you worked with) was beaten, hit, yelled at, raped, threatened, or made feel physical pain for working slowly or for trying to leave?
- Have you ever been forced to work because you felt you were bonded by debt?
- Have you ever been in a situation where you worked in a house that you were not allowed to leave?
- Have you ever worked and someone else was in control of your income?

These four questions elicit the core elements of human trafficking: violence and threats, fictitious debts, confinement, unpaid labour, and control. They were selected to enable the identification of potentially affected individuals during brief encounters in outpatient settings and to facilitate their referral to the study team. For the second group (Group B), a pilot study for a screening for human trafficking was implemented in the reception and registration centre by our research team from December 2021 until June 2022 [26]. Each participant who screened positive for human trafficking was invited to a short confirmatory interview (*N* = 13), which included the questions outlined above, in order to confirm the presence of human trafficking, which was conducted with the help of interpreters if needed. In most cases, either another professional was present to jointly interpret and contextualize experiences of human trafficking through collaborative exchange (*N* = 13), or these experiences were subsequently discussed with an experienced colleague (*N* = 2). Both approaches took part during the COVID-19 pandemic and were therefore subject to restrictions, such as wearing masks, keeping a distance of two metres, and generally heightened awareness of possible infectious diseases. All potential participants were offered the opportunity to contact law enforcement authorities.

Participants were deemed eligible if they met the following criteria: over the age of 18, currently seeking asylum, and positive screening for prior experiences of trafficking victimization, indicated by the screener and a brief follow-up interview. A psychotic or dissociative mental state and acute suicidality were exclusion criteria. Participants were not paid, and participation was completely voluntary. One participant of group A had to be excluded due to an acute psychotic state. Further, two other participants in group A, were excluded after the experiences could not be validated as human trafficking in the personal interview.

### Procedure

After selecting the participants, the interview took place in English, French, or Italian individually in separate rooms. In cases where other languages were spoken (*N* = 5), telephone interpreters were utilized to facilitate communication. The interpreters were professional, independent telephone interpreters (not from the initial reception center), who were informed about the scope and purpose of the interviews at the beginning of each interview. Participants were informed about the study conditions, asked if they agreed to the recording of the interview, and filled out the informed consent sheet. They were thoroughly informed about the voluntary nature and confidentiality of the interview, the independence of the process from any legal proceedings, and their right to withdraw their data or terminate the interview at any time. For the quantitative and descriptive analysis, participants filled out a set of questionnaires in their respective language about their mental health (PHQ-2, GAD-2, PC-PTSD-5, RHS-15 ‘distress thermometer’). They were informed about the relevant outpatient clinics at the initial reception centre where they could receive psychiatric and psychosocial assistance, as well as to social and procedural counselling services, the church representatives within the PHV, and psychological counselling provided by specialized support centers. The semi-structured interview included questions about demographic variables, flight conditions, and the trafficking experience. Before leaving, each participant was thanked for participation and accompanied back to the waiting room in the psychosocial walk-in clinic for refugees (recruitment approach A) or to their accommodation (recruitment approach B).

The interviews were conducted by the first author, who was undergoing further training as a psychological psychotherapist at the time of the study and already had previous experience working with refugees and vulnerable groups. No other support stuff was present during the interviews. As a researcher, the first author approached participants from this vulnerable group with the utmost care, sensitivity, and respect. It is important to acknowledge that many participants had only recently arrived at the initial reception center and remained in a situation of legal uncertainty. Despite providing detailed explanations and assurances regarding anonymity and voluntariness, it is possible that some participants remained sceptical or hesitant to share information. This response was fully respected; no pressure was exerted to answer questions, nor were follow-up inquiries pursued when signs of discomfort were observed. While this may have limited the depth or comprehensiveness of the data, prioritizing the participants’ well-being and autonomy was considered paramount. Furthermore, this study was conducted within a larger research project on human trafficking, during which the first author maintained ongoing and close collaboration with various professional groups working at the initial reception center, all of whom were informed about the study. This continuous exchange fostered a collaborative environment for dialogue and reflection on the experiences and challenges encountered in this context. For all participants the researcher reached out to the local specialised counselling centre for trafficked people, social workers, and the coordinator for accommodation of vulnerable groups within the state reception and registration centre as well as follow-up contact if this was desired.

### Psychometric assessment

The Patient Health Questionnaire 4 (PHQ-4), a 4-item depression and anxiety screening tool, combines the Patient Health Questionnaire 2 (PHQ-2) with a brief version of the Generalized Anxiety Disorder 2 (GAD-2). Response options for all both subscales items are “not at all” (0), “several days” (1), “more than half of the days” (2), and “nearly every day” (3), resulting in a PHQ-2 or GAD-2 total score ranging from 0 to 6. Scores of the subscales ≥ 3 are considered as “yellow flags”, indicating the need for further diagnostic [27].

The Primary Care PTSD Screen for DSM-5 (PC-PTSD-5; [28, 29]) is a checklist designed to identify post-traumatic stress symptoms experienced in the last month. It includes an initial question about experiencing traumatic events. If the individual answers “yes”, the first item is followed by five questions that correspond to the DSM-5 criteria for PTSD (intrusion, internal/external avoidance, hyperarousal and negative alterations in cognitions and mood) which are rated on a binary scale (Yes/No). Higher scores indicate the presence of more post-traumatic symptoms. The total score ranges from 0 to 5. The PC-PTSD-5 scale has demonstrated high sensitivity (0.90-1) and satisfactory specificity (0.67–0.80) when using a total critical cut-off score of ≥ 3 [29, 30].

The Refugee Health Screener-15 (RHS-15; [31]) is a screening tool developed to identify the most common disorders among refugees and asylum seekers. Its “distress thermometer” (DT, item 15) measures perceived distress using a scale ranging from 0 to 10. The RHS-15 authors recommend a cut-off score of ≥ 5 when using the distress thermometer to indicate a positive screening outcome.

### Qualitative and quantitative analysis

All interviews had a duration of twenty to forty-five minutes, including the time needed for interpretation in five cases. The audio file or the translation was transcribed verbatim in English, French, Italian, or German, following predefined transcription rules. The qualitative content analysis was conducted with MAXQDA 2022, Release 22.2.0 [32] following the principles of Mayring [33]. The first author developed a coding framework combining inductive and deductive themes, guided by a review of relevant literature on the support needs of survivors and psychological effects of human trafficking. Each single sentence or pair of content related sentences was assigned to a relevant category as a content analytic unit of analysis. Phrases could be referred to more than one category if they reflected multiple thematic dimensions. The coding—particularly in cases where codes overlapped—was discussed in detail in joint consultation with the co-authors and the research team. The final categories were summarised into main themes until content saturation was achieved [33]. The report of qualitative data follows the Standards for Reporting Qualitative Research (SRQR; [34]). To obtain descriptive statistics, the analysis of demographic variables and psychometric data was managed with Excel, program version 16.84 [35].

## Results

### Sample characteristics

In total, this study includes qualitative interviews with 20 trafficking survivors that applied for asylum in Germany. *N* = 14 participants were female, *N* = 6 participants were male. They were between 20 and 42 years old (Ø 28,05). Further sociodemographic information and the results of the psychometric screening assessment can be found in the two following tables (Tables 1 and 2).

### Key themes

Table 3 demonstrates the key themes and codes that we derived from the semi-structured interviews. We identified 594 statements that were coded and summarised into categories. Finally, eight categories within three main themes were derived.

**Table 1:** Sociodemographic characteristics (*N* = 20)

Region of origin	*N*	%
Gambia	6	30
Cameroon	4	20
Nigeria	4	20
Afghanistan	1	5
Ghana	1	5
Palestine	1	5
Serbia	1	5
Syria	1	5
Turkey	1	5
**Language**	*N*	
English	13	65
Arabic	2	10
French	2	10
Farsi	1	5
Serbian	1	5
Turkish	1	5
**Religion**	*N*	%
Islam	10	50
Christianity	9	45
Atheism	1	5
**Children**	*N*	%
Yes	11	55
No	8	40
No information	1	5
**Current Pregnancy**	7	35
**Type of exploitation** ^1^	*N*	%
Sexual	11	55
Labour	7	35
Trafficked but not exploited^2^	3	15
Forced marriage	1	5
**Time of escape from home country**	Range	
	2009–2023	
**Length of stay in the PHV**	Range	
	5 days – 5 months	

Note. The order is based on the percentages

^1^ double entry is possible

^2^ According to the definition by the UN general assembly in Palermo in 2000 (Office of the United Nations High Commissioner for Human Rights [OHCHR], [1]), the recruitment and transportation of a person for the purpose of exploitation already constitute human trafficking, even if, for example, the person managed to escape during the course of transportation

**Table 2:** Psychometric assessment (*N* = 20)

	M	SD
PHQ-2 *(n = 19)*	3.50	1.82
GAD-2 *(n = 19)*	3.58	1.89
PC-PTSD-5	3.45	1.39
RHS-15 thermometer *(n = 16)*	6.25	3.99

**Table 3:** Key themes and codes

Main themes	Codes	Number of quotes
Trafficking Experience (2)	Form of exploitation Forms of control exerted by traffickers	173 55
Recruitment (2)	Recruitment strategies Rituals	100 31
Exiting trafficking (2)	Escape from trafficking situation Helpers	29 35
Risk Factors and Resilience (2)	Vulnerabilities before being trafficked Personal strength and Resources	101 70

### Theme 1: Recruitment

#### Category 1, ‘Recruitment strategies’ (100 quotes)

Some interviewees (*n* = 7) mentioned that they were in a vulnerable situation, such as due to financial problems or persecution, when someone approached them to lure them into trafficking. Several participants (*n* = 4) explained that a friend of their family or family members themselves suggested to help them and established the contact with the trafficker, meaning that they did not expect a trap. Some were promised work in a shop or as a housekeeper abroad but, when they arrived at their destination, were told to pay back an unrealistic high debt determined for the cost of their flight (*n* = 6). In one case, a man from Serbia belonging to the Roma community reported being promised employment in the construction sector; however, he worked under hazardous conditions and, despite assurances of a good salary, was not paid at all. A young Cameroonian woman was deceived by a man told her that he would adopt her as soon as she arrived in Belgium. Instead, he forced her into sexual exploitation.

*My mother*,* she is dead when I was four or five years. I was still young when my mother’s dead. I… That woman was a friend of my mother (…) Of course*,* I trusted her. Because I was thinking that is a very*,* is a good person - to help me*,* because she was a friend of my mother. And then she took me to do her business. (LC1104*,* Pos. 180–182)*

#### Category 2, ‘Ritual’ (31 quotes)

Some interviewees (*n* = 4, *n* = 3 from Nigeria, *n* = 1 from Gambia) describe they had participated in spiritual rituals (‘juju’) where they had to swear they would not turn against their employers. In this procedure, a female Nigerian participant described how a ‘priest’ took hairs from their armpits, pubic hair, or cut their fingernails, others took photos. Another participant was cut on several parts of her body. Some were told they that they would go crazy because of the juju (*n* = 3) and two persons were told that the juju would kill them. One participant said she felt uncomfortable because part of her body was still with a ‘priest’ in Nigeria. Others (*n* = 7) stated that they had not experienced any rituals and did not believe in charms.

*[They said: ] If I did not obey I’m going to run mad*,* that the juju is going to kill me. (TD1126*,* 187–188)*

*Then I*,* we have to shave my hair*,* here [points to her private parts]. [Yeah]*,* they shave my arm. Take my nails to*,* they have to take my pins. [Mhm] So they say*,* if I don’t pay money back*,* that’s*,* they are*,* the juju is going to kill me. (TD1126*,* 80–83)*

### Theme 2: Trafficking experience

#### Category 1, ‘Forms of exploitation’ (173 quotes)

Five participants reported excessive working hours with payment that was either lower than expected or without payment. Four participants who experienced sex trafficking stated they had no fixed working hours but had to adapt to the arrival of clients, as they were often locked in a house and had to be available at any time. Those who were forced into labour exploitation often worked for more than 12 h during the day (n = 2). All participants were kept in the trafficking situations for different time periods. One female participant from Nigeria reported that she stayed *“five to six days*” (TD1126) with a man who lured her into prostitution in Italy. Another participant from Turkey reported having worked in a restaurant *“for around four years”* (IJ1217) where he was forced to perform sexual acts with the owner and his friends. Other participants reported being trafficked between 2 weeks, several months and up to 4 years. For some of them, like for one woman from Nigeria, it was difficult to remember the time *“In the house*,* I spent few month*,* but I can’t really remember anymore“*(EH1216). Many participants (*n* = 8) mentioned other victims that were forced to work with them, in one case up to 50 people.

*So when it was August*,* then the woman refused to free me*,* she did not even say anything about it. She just keep quiet*,* she just kept quiet*,* I [thought](…) our agreement was August. I*,* I will finish paying the money and then she sets me free*,* she leave me alone. But August she*,* she don’t even say anything*,* she not come to me and say ‘ah*,* you’re through with your payment*,* you can go now’. So I waited August*,* September*,* October*,* no. (IN1219*,* 124)*

*(…) Then he sent me downstairs and assigned me to a machine*,* where I was supposed to do heavier work. For example*,* aluminium or iron or metalworking or something like that. That was heavy work that took longer. (…) And it was exhausting*,* there was exhaustion*,* there was fatigue and there was also a lot of dust and as a result we had to scratch our whole bodies all the time*,* because we came into contact with this dust for the first time. (JV1116*,* 41–45)*

#### Category 2, ‘Forms of control exerted by traffickers’ (55 quotes)

Every participant described some form of violence, insult or threat. In particular, those who were in a vulnerable situation, e.g. undocumented migrants, parents, or a person in need of special medication, obtained threats in order to keep them in the trafficking situation. One mother was separated from her baby son and only allowed to see him a few times a week. One trafficked woman from Gambia was told that, as a black domestic worker, she could not receive medical treatment because she came from another country. Another trafficker prevented medically necessary treatment for a broken arm in order to protect himself. *N* = 10 participants reported experiencing threats or actualized physical violence. These included being beaten, cut with a knife, threatened with weapons, and one person was exposed to electric shocks. A further participant described a horrific scene of what happened to other trafficked people who disobeyed a command.

*She showed me one big ‘drum’ like that. (…) When you refuse to do anything (…) they put you there for maybe three days*,* no food*,* no water. They just allow sun*,* because the Libya sun is so hot. That place the sun will dry you up. (…) It was like a cage*,* outside. (IN1219*,* 98–100)*

*They said if I don’t pay*,* I will stay there forever*,* or they kill me. (FA19*,* 116)*

*They destroy your leg*,* or they will kill you or they will beat you seriously. You can even*,* you can lose your life. So you must go work by*,* you must work by force. (SR1032*,* 186–188)*

*“You are black. Here in Europe when you are black*,* if you go to the place to help they will not help you”* (BD14)

### Theme 3: Exiting trafficking

#### Category 1, ‘Escape from trafficking situation’ (29 quotes)

The participants described different circumstances under which they were able to escape. Some (*N* = 6) waited for a pretext to be allowed to leave the house and turned to the first person they met to ask for help. One male participant described how he just left through a small open window, another female participant escaped through an open door while everyone was sleeping. One Cameroonian survivor of sex-trafficking described that she cried so much that nobody dared to touch her, which is why she was sold by her female trafficker to a Turkish man. She eventually managed to flee from his house and then to Greece with the help of another migrant that paid for her travels. Another Nigerian women managed to escape by verbally objecting to sexual exploitation when they transported her to Libya and then got “kicked out” by her trafficker.

*I was in a closed place; I couldn’t go out (…). When I had my period*,* I told him that I was having my period and that I’d like to have some tampons (…) That’s when he took me to the supermarket*,* and I used his discretion to escape into the street. (MM16*,* 120–121)*

*We were dead anyway*,* so we had nothing to lose*,* so we risked everything. (JO1018*,* 100)*

*And then*,* when I refused she kicked me out of the house at night. And there it was dangerous because I… When we arrived that period*,* every night we heard gunshots. (MR18)*

#### Category 2, ‘Helpers’ (35 quotes)

Several participants (*N* = 9) explained that their escape was only possible because others helped them, either other victims, friends, or someone they just met on the street. Often, they received assistance for nothing in return. Two sexually exploited participants received help by clients that took pity on them. In one case, a Gambian woman was exploited by a family for unpaid cleaning and childcare; a friend of that family later urged them to take her to the hospital. One man from Syria described that some people organized his and his families escape on a boat for free, because they knew that this family had a sick child. One participant was held capture by a paramilitary group in Senegal for years until one day she got the opportunity to escape with a perpetrator who grew tired of his life as a ‘rebel’. He stole the paramilitary group’s vehicle and drove them as far as he could, where their paths parted.

*One day he allowed me to go out*,* because it was close to the sea. I went out*,* I sit in the somewhere there*,* I was crying. And this one African guy*,* he was selling things. He passed there*,* he is a Nigerian guy. He saw me there*,* he asked me what happened*,* why I’m crying. I explain to him. The only thing he tell me is I should be careful*,* because most of girl*,* most of African girl when they come like that*,* later they are missing. And sometimes they find their body in the bush*,* in the water. When they don’t do what they want*,* these Turkish people*,* they can kill you. Nobody will talk*,* who knows you? You have no document. Nobody knows you. So he just tell me to*,* to be careful and to do whatever I want. But if I want he can help me. (LD27*,* 163)*

*So I’m scared the time that boy help me*,* when he tell me: “But you have to go outside and free your mind” I tell: “No*,* I cannot go outside because I’m scared. If I go outside*,* maybe they will kill me like that. He tell me: “No*,* here no one is allowed to do such a thing like that”. I tell: “No*,* I’m the… when the police see me*,* they will arrest me and put me in jail”; he tell me “No*,* they will arrest her… to put in jail.”* (BD14, 166)

### Theme 4: Risk factors and resilience

#### Category 1, ‘Vulnerability before being trafficked’ (101 quotes)

Many participants had lost one or both parents during infancy (*N* = 8) and some grew up in an orphanage (*N* = 2). Consequently, they experienced a high amount of mental distress and were exposed to further risk factors, such as sexual abuse by other caregivers (*N* = 4) or economic hardship (*N* = 4). One Roma participant had been discriminated in his home country in Serbia since his childhood and had difficulty finding a job before he found the ad that lured him into trafficking. General risk factors were wars, armed conflicts, and interpersonal violence which led to the urgent need to leave the country. Two women fled from circumcision, another participant needed to work from early age to support their family. One gay participant from Turkey stated that his sexual orientation and his infection with HIV put him into the dependency of his former employer who exploited him.

*He was in the army for a short time*,* then he got out because he had AIDS. Of course*,* the army discharged him immediately and he was given a pink card*,* which was sent to his family. As soon as his family found out that he had AIDS*,* they immediately rejected him*,* they didn’t want to know anything more about him. In other words*,* he was all alone*,* and now he feels all alone.* (IJ1217, 61, summary by the interpreter)

*So*,* after my dad died*,* after the burial the man came back and said that he need the money. He need the refund back. My mom told him that she don’t have money (…) and she don’t have anywhere to get the money from. So*,* the man say that he was going to take my mum to court*,* he is going to give her problem. So*,* myself and my sister (…) went to see the man and we told him that he should please be patient*,* that we don’t have any money. He said no*,* the only option that we have is that myself get married to him.* (IN1219, 62)

#### Category 2, ‘Personal Strength and Resources’ (70 quotes)

When participants realized that they were in an exploitative situation, four of those interviewed reported experiencing strong internal indignation and an unwillingness to accept it. Three of the interviewees described resisting the intentions of the human traffickers, either through direct verbal confrontation with the individuals involved (*N* = 1) or by forming an internal decision not to remain in the situation (*N* = 2). One participant from Gambia threw away the phone number she was given to call her trafficker as soon as she reached Europe. Many participants said that they have found strength in their religious belief (*N* = 9), of both Christian and Muslim beliefs. Others mentioned their partners and family (*N* = 7), especially their children, that gave them the strength to move on. However, they also mentioned other people (*N* = 4) they got to know, “good people” (SR1932) who provided support. Activities, such as sports, e.g. going to the gym or playing football or education and learning German, were mentioned as helpful, as they distracted them and promoted their well-being (*N* = 8). Others stated they preferred to be on their own sometimes (*N* = 3). Some valued the support they received by networks for migrants and social services (*N* = 5). One Nigerian participant stated that she felt safe in Germany because no one could cause her pain so easily anymore. However, one Afghan woman also stated that she felt that, given everything she had been through, there was nothing that could possibly comfort her.

*So after that*,* the town*,* we meet there*,* I said the work you told me to do – I will not do it (SM30*,* 88)*

*That taught me*,* she said it to me that I have to prostitute. I was filled with anger. That was what brought the courage for me to step up. (MR18*,* 225)*

*I don’t have to talk to [a psychologist]*,* because I believe that that is life*,* if you want to be strong you pass through a lot of challenges. So*,* me I can*,* I believe that any time I read my bible*,* I read some verse and I pray*,* I know that and I*,* I think good. (IN1219*,* Pos. 212)*

*The reason why I take the risk*,* was because of my son. (EH1216*,* 98)*

*I meet with good*,* yeah (?) good*,* good people of*,* in life*,* you know. Anywhere you go*,* you meet good people [Okay] and the good people. So people stand with me. (SR1932*,* 260)*

## Discussion

In this study, twenty trafficking survivors that were accommodated in a reception and registration centre in Germany described their path into trafficking and the violence they had endured. They also portrayed how they escaped and who helped them, as well as how they managed to cope with these experiences. During this difficult time, signs of solidarity and personal strengths were apparent, and religious belief played a crucial role.

Congruent with the existing literature, recruitment frequently occurred in contexts of financial hardship. Such findings are consistent with prior research identifying poverty and financial precarity as some of the most significant risk factors for human trafficking victimization [36, 37]. Severe material deprivation may drive individuals in desperate situations in which exploitative arrangements appear to be the only viable option. Many participants described being lured into trafficking by false promises and deceit, often facilitated by friends or family members who purported to help them escape difficult circumstances—an established pattern in the trafficking literature [38]. Patriarchal structures may further heighten especially women’s vulnerability, as restricted autonomy and the systemic subordination of women can create conditions in which women and girls are steered by members of their own social environment into exploitative situations [36]. Similar findings, namely that poverty or financial emergencies, often compounded by the loss of a caregiver, repeatedly mark the beginning of exploitation and that recruiters are frequently individuals initially trusted by the victims, have been documented in qualitative research with trafficking survivors in Uganda [39]. In the present study, participants’ life circumstances were likewise marked by profound resource deprivation. In many cases, individuals from their close social networks served as the apparent intermediaries who, under the guise of assistance or promising perspectives, established the contacts that ultimately led to exploitation. These situations suggest that individuals’ distress may be exploited even by those within their immediate social circles, underscoring the necessity of social protection and poverty-reduction strategies in trafficking prevention efforts [40].

Spiritual rituals were forced upon several participants, wherein the perpetrators cut hair from their armpits, pubic hair, or cut their fingernails. These rituals called “juju” are practiced in many West African countries and can be used for good or bad purposes. In this sample, three out of four persons who experienced this, were from Nigeria, the other person from the Gambia. Personal items are frequently incorporated as part of the juju—or, as described in the interviews, even materials originating from the body. In cases of human trafficking, it is often the traffickers who perform these juju rituals in order to symbolically impose specific rules or expectations on the individuals concerned [41]. Some of the participants stated, that they still believed in the spell that was supposed to drive them crazy when they turned against the traffickers, which caused extreme levels of anxiety. This often relates to the belief that as long as the personal items or body parts remain with the individual performing the juju, the juju’s power over the affected persons continues to persist [42]. However, there were also other participants who knew about these frightening rituals and strategies to intimidate victims of human trafficking, meaning they were able to distance themselves, so it had no effect on them. This raises the question of whether education about the coercive strategies employed by human traffickers could serve as an effective preventive intervention, or whether the severity of individual hardship would outweigh such measures [9]. According to a study by Donohue-Dioh et al. [43] that interviewed survivors of human trafficking, education and awareness were identified as among the most crucial strategies to prevent individuals from falling victim to human trafficking. Many trafficked persons described that at least one of their parents died during their infancy and several participants had experienced sexualised violence within foster families. Previous studies had already analysed the loss of a parent as a major risk factor for trafficking [20] and physical or sexual abuse as a risk factor for re-trafficking [44]. Other participants experienced poverty and discrimination as member of an ethnic group or LGBTQI*. Besides the loss of attachment figures, armed conflicts and violent cultural customs led to life-threatening events which made the participants more vulnerable to human trafficking.

During the time of exploitation, the participants suffered from verbal, physical, or sexualised violence and endured threats and reprisals. These findings are also corroborated by quantitative studies, in which victims of human trafficking report experiencing physical violence in more than half of all cases [2]. These intimidations during the time of exploitation are well documented and contribute as much as emotional manipulation and an unpredictable environment to high levels of distress [2, 45]. Like a slave, they had to work excessively and received reprisals if they argued against it. Several interviewees, especially those that were sexually exploited, reported the presence of other trafficked persons that were also treated badly. Both the violence described in the interviews and the experiences of coercion and threats were shown to be strongly associated with the development of mental disorders such as post-traumatic stress disorder and depression [46]. As forced migrants represent a group already preoccupied with high levels of psychological stress [47, 48], this results in a twofold mandate: to improve early identification of affected individuals and to provide adequate psychosocial support [49].

To survive the deathly road to Europe as a forced migrant from the Global South, one must display great strength and a will to survive [50]. The need for resilience stretches over different phases of the trafficking experience: In the hands of traffickers, attempts to defend and free themselves were often suppressed by force, but some could escape from the trafficking situation even before they were exploited. Others found creative ways to flee from their perpetrators or asked bystanders for help. In most cases, trafficking survivors rescue themselves, or with the help of other affected individuals, friends, or strangers as anti-trafficking responses fall short [5]. In a qualitative study by Pfeffer et al. [51] factors that facilitate exit from sex trafficking were explored. The findings similarly indicated that survivors primarily relied on support and information from informal networks, made limited use of official or formal assistance services, and that coincidences or critical turning points often played a decisive role in enabling their escape. This raises the question of how identification can be ensured for forced migrants who, for instance, become victims of human trafficking in countries other than their home country, particularly when additional obstacles such as language barriers or lack of legal status are present. However, certain insights into the factors that hinder or facilitate exit from human trafficking may inform policy measures for this population, particularly through educational initiatives and stigma-reduction campaigns implemented within counselling and support services for affected individuals [52].

The participants described occasions in which they showed a particularly high resilience. After arriving in Germany, they described how family, social support, and various activities served as resources for coping with their experiences. Despite the powerlessness of living in a refugee camp, that many saw as obstacle on their road on recovery, the participants knew what improved their well-being. After the exploitation of trust, contact with other people, whether family, partners, or even those they had just met, was an important factor in coping with what they had experienced. A sense of shared identity and solidarity among refugees is known to be a source of support and can possibly mitigate the negative consequences of shared adverse experiences or circumstances [53–55]. Despite its possible risks, asking for help and trusting other refugees and migrants can be a promising source in matters of survival and promote healing in the aftermath of trafficking experiences [54, 56]. Nevertheless, survivors may describe higher levels of mistrust towards others after their trafficking experiences. This can be accompanied by social withdrawal and distance from others for their own protection. Such a supposed ‘protective shell’ ([57]; p. 187) serves to prevent further exploitation and judgement by others but also leads to difficulties in maintaining lasting intimate relationships. This could hinder healing and connectiveness [57]. Often, refugees feel stuck between longing for a meaningful connection with others and the self-imposed intention to keep others at a safe distance. This dynamic may also have influenced the interviews. Some participants may have wished to share their experiences to avoid feeling isolated or to seek help, while others may have been willing to provide only limited information to protect themselves and avoid further uncontrollable risks. Furthermore, recent studies already found that religious belief and interpersonal relationships foster resilience [58, 59]. This supports the notion described by Evans ([54], p. 111) as ‘[t]he experience of trust within relationship was continually attributed as the greatest need of the post-trafficking experience and the primary source of healing and growth’. Initially, the ability to trust put them at risk, but ultimately, it contributed to their recovery. Whether regaining trust succeeds will be decided on an individual level. However, on a structural level, the necessity of trust-building measures, for example at counselling centers, becomes evident. This includes reliability and predictability, personal contact, the flexibility to engage with sometimes challenging life stories, and to endure initial mistrust and scepticism from those affected.

### Limitations

Due to our qualitative approach, this study includes only a small number of participants and is far from representative. It gathers knowledge by the analysis of individual narratives that reflect the personal descriptions of survivors of human trafficking. Nevertheless, this study contributes to the field by including participants who experienced various forms of exploitation - such as labour, domestic servitude, and forced begging - thereby broadening the scope of human trafficking research. This meant that some interviews were conducted via interpreting, which may have created an additional barrier in the mutual understanding between the interviewer and the participants. Furthermore, it should be noted that the quantitative data serve a supplementary function and therefore do not allow for any definitive conclusions regarding the presence of any specific mental illnesses.

## Conclusion

This study targets an especially vulnerable group of trafficking survivors that have experienced, not only human trafficking itself, but also the hardship of flight to Europe. Among the 20 participants, most of whom had experienced sexual or labour exploitation, financial hardship emerged as the central precondition preceding trafficking, corroborating findings from existing research. In addition, several participants from West African countries described being subjected to coercion through spiritual rituals (“juju”), the impact of which persisted for some even after exiting exploitation. Greater awareness and culturally informed understanding of such practices may represent an important component of trafficking prevention efforts. Beyond documenting the substantial burdens and harms endured, it is equally important to highlight the resilience demonstrated by those affected. The findings indicate that most participants ultimately freed themselves from situations of coercion, either through their own agency or with the support of friends or even strangers. Equally important is also the fact that the victims of human trafficking also managed to trust someone, despite the breach of trust that brought them into the situation in the first place. Social relationships, including ties to family, children, and friends, alongside religious faith emerged as central resources in coping with and processing adverse experiences. These findings underscore that survivors of trafficking should not be understood solely through the lens of victimization, but also as agents who mobilize personal and social resources to reclaim autonomy and shape their futures.

## Data Availability

The data that support the findings of this study are available on reasonable request from the corresponding author, IRM. The data are not publicly available due to ethical considerations.

## References

[1] Office of the United Nations High Commissioner for Human Rights (OHCHR). Protocol to prevent, suppress and punish trafficking in persons, especially women and children, supplementing the United Nations Convention against transnational organized crime. New York: United Nations; 2000. (GA Res 55/25, Annex II, UN GAOR, 55th Sess, Supp No 49, UN Doc A/45/49/Vol I).

[2] Stöckl H, Fabbri C, Cook H, Galez-Davis C, Grant N, Lo Y, Kiss L, Zimmerman C. Human trafficking and violence: Findings from the largest global dataset of trafficking survivors. J Migration Health. 2021;4:100073. 10.1016/j.jmh.2021.100073. 34888537 10.1016/j.jmh.2021.100073 PMC8637135

[3] International Labour Organization (ILO). Human Trafficking and Forced Labour Exploitation Guidelines for Legislation and Law Enforcement. In. Geneva: International Labour Organization; 2005.

[4] Wheaton EM, Schauer EJ, Galli TV. Economics of human trafficking. Int migration. 2010;48(4):114–41. 10.1111/j.1468-2435.2009.00592.x 20645472

[5] United Nations Office on Drugs and Crime (UNODC). Global report on trafficking in persons 2022. Vienna: United Nations Office on Drugs and Crime; 2023. (Sales No. E.23.IV.3).

[6] García-Vázquez O, Meneses-Falcón C. What is the Impact of Human Trafficking on the Biopsychosocial Health of Victims: A Systematic Review. J Immigr Minor Health. 2024;26(1):148–62. 10.1007/s10903-023-01496-z. 37222868 10.1007/s10903-023-01496-z

[7] Zimmerman C, Kiss L. Human trafficking and exploitation: A global health concern. PLoS Med. 2017;14(11):e1002437. 29166396 10.1371/journal.pmed.1002437 PMC5699819

[8] International Labour Organization (ILO), Walk Free Foundation, International Organization for Migration (IOM). Global estimates of modern slavery: forced labour and forced marriage. Geneva: International Labour Organization; 2022. https://www.ilo.org/publications/major-publications/global-estimates-modern-slavery-forced-labour-and-forced-marriage.

[9] Latham-Sprinkle J, David F, Bryant K, Larsen J. Migrants and their vulnerability to human trafficking, modern slavery and forced labour. Geneva: International Organization for Migration; 2019.

[10] United Nations Office on Drugs and Crime (UNODC). Global report on trafficking in persons 2020. Vienna: United Nations Office on Drugs and Crime; 2021. (Sales No. E.20.IV.3).

[11] Wilson A. Trafficking risks for refugees. Soc Without Borders. 2012;7(1):100–18.

[12] United Nations Office on Drugs and Crime [UNODC]. Global Report on Trafficking in Persons 2024. In. Vienna: United Nations Office on Drugs and Crime [UNODC]; 2024.

[13] Gezie LD, Yalew AW, Gete YK. Human trafficking among Ethiopian returnees: its magnitude and risk factors. BMC Public Health. 2019;19(1):104. 10.1186/s12889-019-6395-z. 30669989 10.1186/s12889-019-6395-z PMC6343328

[14] Brunovskis A, Surtees R. Vulnerability and exploitation along the Balkan route: Identifying victims of human trafficking in Serbia. Oslo: FAFO, Nexus, Atina & CYI; 2017.

[15] Nkwanzi V, Bulamba RM, Okech D, Evans D, Akello D, Kyasanku E, Daama A, Mugamba S, Namakula S, Carlson C, Nalugoda F, Kigozi G, Nakigozi G. Individual and Communal Factors Associated with Re-Trafficking Vulnerability Among Women Survivors in Kampala, Central Uganda. J interpers Violence. 2025;0(0):08862605251392277. 10.1177/08862605251392277. 10.1177/08862605251392277 41454625

[16] Vella S-LC, Pai NB. A Theoretical Review of Psychological Resilience: Defining Resilience and Resilience Research over the Decades. Archives Med Health Sci. 2019;7(2):233–9. 10.4103/amhs.amhs_119_19.

[17] Windle G. What is resilience? A review and concept analysis. Reviews Clin Gerontol. 2011;21(2):152–69. 10.1017/S0959259810000420.

[18] Weitzel EC, Glaesmer H, Hinz A, Zeynalova S, Henger S, Engel C, Löffler M, Reyes N, Wirkner K, Witte AV, Villringer A, Riedel-Heller SG, Löbner M. What Builds Resilience? Sociodemographic and Social Correlates in the Population-Based LIFE-Adult-Study. Int J Environ Res Public Health. 2022;19(15):9601. https://www.mdpi.com/1660-4601/19/15/9601. 35954965 10.3390/ijerph19159601 PMC9368156

[19] McGee SL, Höltge J, Maercker A, Thoma MV. Sense of coherence and stress-related resilience: investigating the mediating and moderating mechanisms in the development of resilience following stress or adversity. Front Psychiatry. 2018;9. 10.3389/fpsyt.2018.00378. 10.3389/fpsyt.2018.00378 PMC6110848 30186189

[20] Knight L, Xin Y, Mengo C. A scoping review of resilience in survivors of human trafficking. Trauma Violence Abuse. 2022;23(4):1048–1062. 10.1177/1524838020985561. 10.1177/1524838020985561 33468034

[21] Knight L, Ploss A, Benavides J, Yoon S. A Qualitative Study of Risk and Protective Factors for Resilience in Survivors of Sex Trafficking. Violence Against Women. 2023;29(15–16):3302–24. 10.1177/10778012231192587. 37545376 10.1177/10778012231192587

[22] Knight L, Yoon S. You come up from the ashes, and you’re like a phoenix. Survivors of sex trafficking define resilience. Qualitative Social Work. 2024;23(3):424–41. 10.1177/14733250231152263.

[23] Manok N, Huhn D, Kohl RM, Ludwig M, Schweitzer J, Kaufmann C, Terhoeven V, Ditzen B, Herpertz SC, Herzog W, Nikendei C. Ambulanz für Geflüchtete mit Traumafolgestörungen und psychischen Belastungen in einer Landeserstaufnahmeeinrichtung. Psychotherapeut. 2017;62(4):333–40. 10.1007/s00278-017-0205-9.

[24] Nikendei C, Huhn D, Adler G, von Rosen P, Eckstein TM, Fuchs B, et al. Entwicklung und Implementierung einer medizinischen Ambulanz in einer Erstaufnahmeeinrichtung für Asylsuchende des Landes Baden-Württemberg. Z Evid Fortbild Qual Gesundhwes. 2017;126:31–42. 10.1016/j.zefq.2017.07.011. 10.1016/j.zefq.2017.07.011 28935457

[25] Zehetmair C, Zeyher V, Cranz A, Ditzen B, Herpertz SC, Kohl RM, Nikendei C. A walk-in clinic for newly arrived mentally burdened refugees: the patient perspective. Int J Environ Res Public Health. 2021;18(5):2275. https://www.mdpi.com/1660-4601/18/5/2275. 10.3390/ijerph18052275 PMC7956492 33668936

[26] Tambini Stollwerck EA, Rollmann I, Friederich H-C, Nikendei C. Responding to human trafficking among refugees: prevalence and test accuracy of a modified version of the adult human trafficking screening tool. BMC Public Health. 2024;24(1):1685. 10.1186/s12889-024-18997-7. 38914998 10.1186/s12889-024-18997-7 PMC11197328

[27] Löwe B, Wahl I, Rose M, Spitzer C, Glaesmer H, Wingenfeld K, Schneider A, Brähler E. A 4-item measure of depression and anxiety: Validation and standardization of the Patient Health Questionnaire-4 (PHQ-4) in the general population. J Affect Disord. 2010;122(1):86–95. 10.1016/j.jad.2009.06.019. 19616305 10.1016/j.jad.2009.06.019

[28] Prins A, Bovin MJ, Kimerling R, Kaloupek DG, Marx BP, Pless Kaiser A, et al. The Primary Care PTSD Screen for DSM-5 (PC-PTSD-5) [measurement instrument]. Bielefeld (Germany): Universität Bielefeld; 2015.

[29] Prins A, Bovin MJ, Smolenski DJ, Marx BP, Kimerling R, Jenkins-Guarnieri MA, et al. The primary care PTSD screen for DSM-5 (PC-PTSD-5): development and evaluation within a veteran primary care sample. J Gen Intern Med. 2016;31(10):1206–11. 10.1007/s11606-016-3703-5 PMC5023594 27170304

[30] Williamson ML, Stickley MM, Armstrong TW, Jackson K, Console K. Diagnostic accuracy of the Primary Care PTSD Screen for DSM-5 (PC-PTSD-5) within a civilian primary care sample. J Clin Psychol. 2022;78(11):2299–308. 10.1002/jclp.23405 35763419

[31] Hollifield M, Verbillis-Kolp S, Farmer B, Toolson EC, Woldehaimanot T, Yamazaki J, et al. The refugee health screener-15 (RHS-15): development andvalidation of an instrument for anxiety, depression, and PTSD in refugees. Gen Hosp Psychiatry. 2013;35(2):202–9. 10.1016/j.genhosppsych.2012.12.002 23347455

[32] VERBI Software. MAXQDA: software for qualitative data analysis. Berlin: VERBI Software; 2022. Available from: https://www.maxqda.com. Accessed 6 May 2026.

[33] Mayring P. Qualitative Inhaltsanalyse. Weinheim: Beltz Verlagsgruppe; 2015. http://www.content-select.com/index.php?id=bib_view&ean=9783407293930. https://content-select.com/de/portal/media/view/552557d1-12fc-4367-a17f-4cc3b0dd2d03.

[34] O’Brien BC, Harris IB, Beckman TJ, Reed DA, Cook DA. Standards for Reporting Qualitative Research: A Synthesis of Recommendations. Acad Med. 2014;89(9):1245–51. 10.1097/acm.0000000000000388. 24979285 10.1097/ACM.0000000000000388

[35] Microsoft Corporation. Microsoft Excel [computer program]. Redmond (WA): Microsoft Corporation; 2024. https://office.microsoft.com/excel.

[36] Islam F, Khan MA. Exploring the socio-economic vulnerability model: a multi-stakeholder analysis of women’s trafficking in Bangladesh. Cogent Social Sci. 2025;11(1):2549477. 10.1080/23311886.2025.2549477.

[37] Sharapov K, Komenda H. Conceptualising Vulnerability in Forced Displacement: The Role of Human Rights-Based Approaches in Mitigating Trafficking Risks—Findings From Poland and Romania. Int migration. 2025;63(5):e70081. 10.1111/imig.70081.

[38] Shepherd DA, Parida V, Williams T, Wincent J. Organizing the exploitation of vulnerable people: A qualitative assessment of human trafficking. J Manag. 2022;48(8):2421–57.

[39] Klabbers RE, Hughes A, Dank M, O’Laughlin KN, Rogers M, Stoklosa H. Human trafficking risk factors, health impacts, and opportunities for intervention in Uganda: a qualitative analysis. Global Health Res Policy. 2023;8(1):52. 10.1186/s41256-023-00332-z. 10.1186/s41256-023-00332-z PMC10712038 38072964

[40] Perry KM, McEwing L. How do social determinants affect human trafficking in Southeast Asia, and what can we do about it? A systematic review. Health Hum Rts. 2013;15:138. 24421161

[41] Adeyinka S, Lietaert I, Derluyn I. The Role of Juju Rituals in Human Trafficking of Nigerians: A Tool of Enslavement, But Also Escape. Sage Open. 2023;13(4):21582440231210474. 10.1177/21582440231210474.

[42] Nagle LE, Owasanoye B. Fearing the dark: The use of witchcraft to control human trafficking victims and sustain vulnerability. Sw L Rev. 2015;45:561.

[43] Donohue-Dioh J, Otis M, Miller JJ, Sossou M-A, delaTorres C, Lawson T. Survivors’ conceptualizations of human trafficking prevention; An exploratory study. Eval Program Plan. 2020;83:101873. 10.1016/j.evalprogplan.2020.101873. 10.1016/j.evalprogplan.2020.101873 33157374

[44] Chen VH, Beauchemin EL, Cuan IT, Sadana A, Olulola-Charles L, Leschi JE, Ades V. Sex Trafficking in New York City and Vulnerabilities to Re-Trafficking. J interpers Violence. 2023;38(21–22):11501–19. 10.1177/08862605231183452. 37421223 10.1177/08862605231183452

[45] Zimmerman C, Yun K, Shvab I, Watts C, Trappolin L, Treppete M, Bimbi F, Adams B, Jiraporn ST, Beci L. The health risks and consequences of trafficking in women and adolescents: findings from a European study. London: London School of Hygiene & Tropical Medicine; 2003.

[46] Iglesias-Rios L, Harlow SD, Burgard SA, Kiss L, Zimmerman C. Mental health, violence and psychological coercion among female and male trafficking survivors in the greater Mekong sub-region: a cross-sectional study. BMC Psychol. 2018;6(1):56. 10.1186/s40359-018-0269-5. 30541612 10.1186/s40359-018-0269-5 PMC6292017

[47] Blackmore R, Boyle JA, Fazel M, Ranasinha S, Gray KM, Fitzgerald G, Misso M, Gibson-Helm M. The prevalence of mental illness in refugees and asylum seekers: A systematic review and meta-analysis. PLoS Med. 2020;17(9):e1003337. 10.1371/journal.pmed.1003337. 32956381 10.1371/journal.pmed.1003337 PMC7505461

[48] Patanè M, Ghane S, Karyotaki E, Cuijpers P, Schoonmade L, Tarsitani L, Sijbrandij M. Prevalence of mental disorders in refugees and asylum seekers: a systematic review and meta-analysis. Global Mental Health. 2022;9:250–63. 10.1017/gmh.2022.29. 36618716 10.1017/gmh.2022.29 PMC9806970

[49] Tambini Stollwerck EA, Friederich H-C, Nikendei C. Menschenhandel im Kontext von Flucht und Vertreibung: Definition, psychische Belastung und Implikationen für die psychosoziale Versorgung. Psychother Psychosom Med Psychol. 2022;72(1):58–68. 10.21706/tg-16-1-58.

[50] Black J. A decade of documenting migrant deaths: data analysis and reflection on deaths during migration documented by IOM’s Missing Migrants Project, 2014–2023. Berlin: International Organization for Migration, Global Migration Data Analysis Centre (IOM GMDAC); 2024. https://missingmigrants.iom.int/sites/g/files/tmzbdl601/files/publication/file/A%20decade%20of%20documenting%20migrant%20deaths.pdf.

[51] Pfeffer R, Barrick K, Galvan T. Barriers and facilitators to leaving a trafficker: A qualitative analysis of the accounts of people who have experienced sex trafficking. Victims Offenders. 2024;19(8):1451–70.

[52] Ferrari C. The factors involved in the exit from sex trafficking: A review. J Int women’s Stud. 2021;22(5):195–209.

[53] Alfadhli K, Drury J. The role of shared social identity in mutual support among refugees of conflict: An ethnographic study of Syrian refugees in Jordan. J Community Appl Social Psychol. 2018;28(3):142–55.

[54] Evans H. The integral role of relationships in experiences of complex trauma in sex trafficking survivors. Int J Hum Rights Healthc. 2018;13(2):109–23. 10.1108/IJHRH-07-2019-0054.

[55] Woods K. Refugees’ Stories: Empathy, Agency, and Solidarity. J Soc Philos. 2020;51(4):507–25. 10.1111/josp.12307.

[56] Saul J, Simon W. Building Resilience in Families, Communities, and Organizations: A Training Program in Global Mental Health and Psychosocial Support. Fam Process. 2016;55(4):689–99. 10.1111/famp.12248. 27578291 10.1111/famp.12248

[57] Mumey A, Sardana S, Richardson-Vejlgaard R, Akinsulure-Smith AM. Mental health needs of sex trafficking survivors in New York City: Reflections on exploitation, coping, and recovery. Psychological Trauma: Theory, Research. Pract Policy. 2021;13(2):185–92. 10.1037/tra0000603. 10.1037/tra0000603 33119348

[58] Luthar SS, Zelazo LB. Research on resilience: an integrative review. In: Luthar SS, editor. Resilience and vulnerability: adaptation in the context of childhood adversities. Cambridge: Cambridge University Press; 2003. p. 510–549. 10.1017/CBO9780511615788.023.

[59] Prati G, Pietrantoni L. Optimism, social support, and coping strategies as factors contributing to posttraumatic growth: a meta-analysis. J Loss Trauma. 2009;14(5):364–388. 10.1080/15325020902724271.

